# Carriage Rate and Effects of Vaccination after Outbreaks of Serogroup C Meningococcal Disease, Brazil, 2010

**DOI:** 10.3201/eid2005.130948

**Published:** 2014-05

**Authors:** Marco Aurelio Palazzi Sáfadi, Telma Regina Marques Pinto Carvalhanas, Ana Paula de Lemos, Maria Cecilia Outeiro Gorla, Maristela Salgado, Lucila O. Fukasawa, Maria Gisele Gonçalves, Fabio Higa, Maria Cristina Cunto Brandileone, Claudio Tavares Sacchi, Ana Freitas Ribeiro, Helena Keico Sato, Lucia Ferro Bricks, José Cassio de Moraes

**Affiliations:** Faculdade de Ciências Médicas da Santa Casa, São Paulo, Brazil (M.A.P. Sáfadi, J.C. de Moraes);; Centro de Vigilância Epidemiológica Alexandre Vranjac, São Paulo (T.R.M.P. Carvalhanas, A.F. Ribeiro, H.K. Sato);; Instituto Adolfo Lutz, São Paulo (A.P. de Lemos, M.C.O. Gorla, M. Salgado, M.G. Gonçalves, L.O. Fukasawa, F. Higa, M.C.C. Brandileone, C.T. Sacchi);; Sanofi Pasteur Vaccines, São Paulo (L.F. Bricks)

**Keywords:** meningococcal carriage, meningococcal vaccines, meningococcus, Neisseria meningitidis, herd immunity, bacteria, vaccination, Brazil, transmission, epidemic strain, serogroup C

## Abstract

During 2010, outbreaks of serogroup C meningococcal (MenC) disease occurred in 2 oil refineries in São Paulo State, Brazil, leading to mass vaccination of employees at 1 refinery with a meningococcal polysaccharide A/C vaccine. A cross-sectional study was conducted to assess the prevalence of meningococci carriage among workers at both refineries and to investigate the effect of vaccination on and the risk factors for pharyngeal carriage of meningococci. Among the vaccinated and nonvaccinated workers, rates of overall meningococci carriage (21.4% and 21.6%, respectively) and of MenC carriage (6.3% and 4.9%, respectively) were similar. However, a MenC strain belonging to the sequence type103 complex predominated and was responsible for the increased incidence of meningococcal disease in Brazil. A low education level was associated with higher risk of meningococci carriage. Polysaccharide vaccination did not affect carriage or interrupt transmission of the epidemic strain. These findings will help inform future vaccination strategies.

## Abstract 2

Polysaccharide vaccine did not affect carriage nor interrupt transmission of an epidemic strain.

In Brazil, meningococcal disease is endemic; 1.5–2.0 cases per 100,000 inhabitants were reported during 2000–2009. Since 2002, a substantial increase has been observed in the proportion of cases attributed to meningococcus serogroup C (MenC) that is associated with the sequence type (ST) 103 complex, and MenC is currently responsible for most cases of meningococcal disease in Brazil (*1*–*3*).

Several outbreaks of MenC disease have been reported in Brazil in recent years (*2*,*4*–*6*). To control these outbreaks, chemoprophylaxis is administered to contacts of infected persons, and vaccination is often recommended for persons in age groups at higher risk for infection. In these reactive vaccination campaigns, meningococcal C conjugate (MCC) vaccine use is restricted to children <2 years of age because of cost and supply issues; meningococcal A/C polysaccharide vaccine is recommended for persons >2 years of age (*1*–*3*).

Published data describing meningococci carriage in Brazil are limited. Few studies have been conducted that assess 1) the role of carriage prevalence in the dynamics of carriage and disease or 2) the potential effect of control programs, such as vaccination, on the transmission of meningococci. Thus, we conducted a cross-sectional study with the primary objective of assessing the prevalence of meningococcal carriage among workers at 2 oil refineries in São Paulo State, Brazil, where outbreaks of MenC disease occurred in 2010. We also investigated the effect of meningococcal A/C polysaccharide vaccination and risk factors on pharyngeal carriage of meningococci.

## Methods

During March 29–June 30, 2010, an outbreak of MenC disease, associated with the ST103 complex, occurred in an oil refinery (Refinery A) with 17,590 workers in São Paulo State, Brazil. A total of 18 cases and 3 deaths (case-fatality rate 16.7%) were associated with the outbreak. Six of the cases and 2 deaths involved Refinery A workers, and 12 of the cases and 1 death involved contacts (family members) of the refinery workers. The case-patients were residents of Cosmópolis, a municipality with 59,000 inhabitants located near Refinery A.

On March 29, health authorities were notified of the first 3 case-patients (2 adult workers at Refinery A and an 8-month-old child whose father worked at Refinery A). An investigation was initiated, and chemoprophylaxis with rifampin was recommended for all close contacts of the 3 index case-patients. During the following 2 weeks, 5 new cases of MenC disease were identified (3 in Refinery A workers and 2 in children who were relatives of Refinery A workers). With these new cases, the incidence of meningococcal disease reached 34.1 cases/100,000 persons at Refinery A. Meningococcal A/C polysaccharide vaccination was recommended for all 17,590 workers at Refinery A. Vaccination began on April 16, and 1 week later, 91% coverage of workers at Refinery A was achieved. However, despite the vaccination program, 10 new cases of MenC disease occurred: 9 cases were in family contacts and 1 case was in a Refinery A worker who had received vaccine 1 day before symptom onset.

The incidence of MenC disease in Cosmópolis subsequently reached 20.2 cases/100,000 persons. Cases occurred in relatives (8 months to 16 years of age) of Refinery A workers, prompting a mass vaccination of 18,571 inhabitants of Cosmópolis who were 2 months to 19 years of age. Vaccination began on June 30, and 90.5% coverage was achieved 1 week later. Infants and toddlers received MCC vaccine, and persons 2–19 years of age received meningococcal A/C polysaccharide vaccine. In the months following the vaccination campaign, no more MenC cases were reported, and the outbreak was considered controlled.

The second outbreak of MenC disease occurred in a refinery with 16,000 workers (Refinery B) in São José dos Campos, a city with 610,095 inhabitants in São Paulo State. On July 10, 2010, a worker at Refinery B was reported to have MenC disease, and on July 18, a second worker was reported to be infected. An investigation identified 10 other reported cases in São José dos Campos during April–July, 2010; the 10 cases were in children <4 years of age who were household contacts of Refinery B workers. Of the 12 identified case-patients, 6 died. As in Cosmópolis, these initial cases were considered the index cases. In Refinery B, the incidence of meningococcal disease reached 12.5 cases/100,000 persons, and a decision was made to provide chemoprophylaxis, but not vaccine, to all close contacts of index case-patients. On August 8, 1 new case of meningococcal disease was reported in a family contact of a Refinery B worker; no further cases were reported in 2010.

Beginning in December 2010, we conducted a cross-sectional study of 483 workers (18–39 years of age) from Refinery A, where mass vaccination had been recommended, and Refinery B, where mass vaccination had not been advised. All study participants gave informed consent. A questionnaire was used to obtain information regarding age, sex, recent respiratory tract infections, active and passive smoking, alcohol consumption, recent antimicrobial drug use, length of employment at the refinery, number of household members living in the same room, level of education, and meningococcal A/C polysaccharide vaccination status.

### Specimen Collection

During the first 2 weeks of December, 2010, we obtained oropharyngeal swab samples from 483 refinery workers (238 vaccinated workers from Refinery A and 245 nonvaccinated workers from Refinery B). The samples were immediately put into transport medium (*7*) and, within 4–5 h, sent to the Adolfo Lutz Institute (São Paulo, Brazil), the National Reference Laboratory for Bacterial Meningitis, where they were stored until use. The stored oropharyngeal swabs were plated onto selective medium, and after 24–48 h of incubation at 37°C (±2°) in 5% CO_2_, the samples were inspected. Samples with meningococcus-like colonies were subcultured on blood agar medium for species identification. Isolates identified as *Neisseria meningitidis* were serogrouped by using an agglutination test. Antisera were obtained for serogroups A, B, C, E, W, X, Y, and Z (*8*,*9*).

### DNA Extraction and Real-Time PCR

DNA from each sample was extracted and purified by using the QIAamp DNA MiniKit (QIAGEN, Alameda, CA, USA) or a similar testing kit according to the manufacturer’s instructions. Primers and fluorescent probes were used for the detection of *N. meningitidis* *ctrA* (*10*) and *sodC* genes by real-time PCR (*11*). Samples positive for *N. meningitidis* were genotyped by using primers and fluorescent probes for *N. meningitidis* serogroups A, B, C, W, and X.

### Serotyping and Multilocus Sequence Typing

Serotyping for all *N. meningitidis* isolates was performed by dot blot analysis, using whole-cell suspensions as described (*12*). Multilocus sequence typing was performed according to the methods of Maiden et al. (*13*). Primers, determination of sequence alleles, and designation of sequence types are described on the Neisseria Multi Locus Sequence Typing website (http://neisseria.org/nm/typing/mlst).

### Statistical Analyses

Using an estimate that the prevalence of meningococci carriage among adults would be ≈18% (±5%), we calculated that ≈225 study participants from each refinery would be needed to analyze all variables. Demographic data for all participants and typing results of *N. meningitidis* isolates were entered into an EpiInfo database (wwwn.cdc.gov/epiinfo/) and compared by using the 2-sided Fisher exact test. Assessment of risk factors was performed using Fisher exact test.

## Results

Of the 483 oropharyngeal samples tested, 104 (21.5%; 95% CI 18.0%–25.5%) were positive for meningococci. Carriage rates were similar among workers from both refineries (21.4% vs. 21.6%). Of the 104 positive samples, 95 were detected by culture and real-time PCR, 1 was detected by culture only, and 8 were detected by real-time PCR only.

The serogroup and genogoup could be determined for 56 of the 104 meningococci-positive samples: 27 (48.2%) were serogroup C, 9 (16.1%) serogroup B, 8 (14.3%) serogroup E, 7 (12.5%) serogroup Y, and 5 (8.9%) serogroup W. The serogroup could not be determined for 48 (46.1%) isolates. The difference in MenC carriage rates among workers at the 2 refineries was not significant: 6.3% at Refinery A and 4.9% at Refinery B (p = 0.48) (Table 1).

**Table 1:** Pharyngeal carriage of *Neisseria meningitidis* among vaccinated and nonvaccinated workers at 2 oil refineries, São Paulo, Brazil, 2010*

*N. meningitidis* serogroup	No. (%) workers			p value‡
	Refinery A*	Refinery B†	Total	
All	51 (21.4)	53 (21.6)	104 (21.5)	1.00
C	15 (6.3)	12 (4.9)	27 (5.6)	0.64
B	4 (1.6)	5 (2.0)	9 (1.9)	1.00
W	4 (1.6)	1 (0.4)	5 (1.0)	0.35
Y	5 (2.1)	2 (0.8)	7 (1.4)	0.43
E	3 (1.2)	5 (2.0)	8 (1.7)	0.76
Nongroupable	20 (8.4)	28 (11.4)	48 (9.9)	0.34
Negative	187 (78.6)	192 (78.4)	379 (78.5)	
Total	238 (100.0)	245 (100.0)	483 (100.0)	

*Vaccinated workers. †Unvaccinated workers. **‡**By Fisher exact test.

### Serotyping and Multilocus Sequence Typing

A total of 38 different serotype–serosubtype antigen combinations were identified among the 96 *N. meningitidis* isolates. Among MenC isolates, phenotype C:23:P.14–6 was the most prevalent (10/13 [77%]). Eleven different STs were found among 27 isolates characterized by multilocus sequence typing. The 11 STs were grouped into 6 different clonal complexes: ST103 complex (n = 7), ST11 complex (n = 5), ST213 complex (n = 1), ST32 complex (n = 3), ST41/44/Lineage 3 (n = 1), ST461 (n = 1). The most prevalent clonal complex, ST103 complex, was represented by ST3780 (n = 6) (Table 2).

**Table 2:** Phenotypic and genotypic characteristics of *Neisseria meningitidis* strains isolated from nasopharyngeal samples of workers at 2 oil refineries, São Paulo, Brazil, 2010*

Refinery, *N. meningitidis* serogroup, worker’s age, y	Serotype:serosubtype	ST	Clonal complex
A			
B			
20	4,7:NST	9858	
29	19,1:P1.14	6481	ST213 complex
19	17:P1.5	8035	ST41/44 complex/Lineage 3
C			
26	23:P1.14–6	3780	ST103 complex
28	23:P1.14–6	8730	NA
21	23:P1.14–6	8730	NA
22	23:P1.14–6	8730	NA
20	23:P1.14–6	8730	NA
W			
21	2b:P1.2	11	ST11 complex/ET-37 complex
27	2b:P1.5,2	11	ST11 complex/ET-37 complex
24	2b:P1.5,2	11	ST11 complex/ET-37 complex
Y			
23	2a:P1.5,2	11	ST11 complex/ET-37 complex
26	17,7:P1.5	6525	NA
22	17,7:P1.5	6525	NA
B			
B			
26	19,1:NST	1869	ST461 complex
19	4,7:P1.19,15	7594	ST32 complex/ET-5 complex
28	4,7:P1.19,15	7594	ST32 complex/ET-5 complex
C			
25	23:P1.14–6	3780	ST103 complex
21	23:P1.14–6	3780	ST103 complex
25	23:NST	3780	ST103 complex
28	23:P1.5	3779	ST103 complex
23	4,7:P1.19,15	3773	ST32 complex/ET-5 complex
28	23:P1.14–6	3780	ST103 complex
25	23:P1.14–6	8730	NA
23	23:P1.14–6	3780	ST103 complex
W			
26	2b:P1.2	11	ST11 complex/ET-37 complex
Y			
19	19,7:P1.5	6525	NA

*NST, not serosubtypeable; ST, sequence type; NA, assigned without clonal complex.

We did not find an increased risk of meningococci carriage associated with any of the potential risk factors studied, except low level of education. A low education level (i.e., not completing secondary education) was significantly associated with a higher risk for carriage of meningococci, regardless of serogroup identification (Table 3).

**Table 3:** Risk factors for pharyngeal carriage of *Neisseria meningitidis* among workers at 2 oil refineries, São Paulo, Brazil, 2010

Variable	All *N. meningitidis* strains				Serogrouped *N. meningitidis* strains		
	% Workers exposed	% Workers not exposed	p value*		% Workers exposed	% Workers not exposed	p value*
Antimicrobial drug use†	12.9	22.1	0.16		3.2	12.0	0.11
Crowded living conditions	17.4	22.9	0.14		9.9	12.3	0.35
Active smoking	23.2	21.2	0.41		11.6	11.6	0.58
Respiratory symptoms†	24.2	20.9	0.26		10.1	11.9	0.44
Low level of education‡	32.9	19.2	0.01		17.0	10.6	0.07

*By Fisher exact test. †In the 15 d before the collection of the nasopharyngeal sample. ‡Defined as not completing secondary education.

## Conclusions

Most published studies report a consistently low rate (usually <1%) of MenC carriage during outbreaks of MenC disease (*14*–*16*). However, after outbreaks at 2 oil refineries in São Paulo State, Brazil, we found high rates (6.3% and 4.9%, respectively) of MenC carriage among refinery workers.

Mass vaccination with a meningococcal A/C polysaccharide vaccine was conducted at Refinery A, and high coverage (91%) was achieved among workers. This intervention controlled the MenC outbreak in the refinery; only 1 new case occurred after the vaccination campaign, but that case cannot be considered the result of a vaccine failure because it occurred <14 days after the refinery worker was vaccinated. These findings likely indicate that the workers received direct protection against MenC from vaccine. However, after the vaccination campaign, 9 new cases of MenC infection occurred in children who were household contacts of vaccinated workers, without any known contact among them.

The prevalence of MenC carriage was high among workers at both refineries, even though 91% of Refinery A workers had received meningococcal A/C polysaccharide vaccine 6 months before our study began. More striking, carriage rates among vaccinated and nonvaccinated workers were similar. These findings suggest that meningococcal A/C polysaccharide vaccine had no effect on MenC carriage. Most of the studies conducted among nonmilitary populations demonstrated that these vaccines cannot significantly reduce meningococcal carriage (*17*–*20*). The short-term persistence of circulating antibodies and the quality of the immune response induced after vaccination with a polysaccharide vaccine may partly explain why these vaccines have no effect on carriage (*20*–*24*).

In contrast to polysaccharide vaccines, conjugate vaccines lead to the production of very high antibody concentrations, even in infants, and induce immunologic memory with higher antibody avidity and increased serum bactericidal activity, thus providing more robust long-term protection. In addition, conjugate vaccines also prevent the acquisition of carriage among vaccinees and, by interrupting transmission, provide indirect protection to unvaccinated, susceptible persons; this herd immunity proved key to the success of MCC vaccination programs in various countries (*25*–*27*).

The characterization of the *N. meningitidis* strains isolated from the patients (workers and family contacts) during the outbreak in Refinery A has been described (*28*). The characterization showed that all MenC isolates were genetically related and displayed the same phenotype, C:23:P1.14–6, associated with ST3780 of the ST103 complex. The characterization of the 13 MenC carriage strains recovered from workers at both refineries in our study showed that most (10/13) displayed the C:23:P1.14–6 phenotype. These strains displayed 2 STs: ST3780, which belongs to ST103 complex, and ST8730, assigned without clonal complex. In Brazil, the increase in MenC disease during the last decade has been associated with the emergence of this virulent clone belonging to the ST103 complex (*2*,*29*). The ability of MCC vaccines to effect carriage of strains from the ST103 complex has yet to be shown. The recent introduction of MCC vaccine in the routine immunization program in Brazil will provide this opportunity, highlighting the importance of carefully designed studies to measure the effect of the vaccine on carriage and transmission.

Meningococcal carriage was not associated with any of the risk factors evaluated in our study, except the level of education, which was inversely related to the prevalence of carriage. The higher percentage of MenC carriers among study participants with a lower level of education presumably reflects associated socioeconomic conditions and social behaviors. Less-educated workers in oil refinery settings are also more likely to perform activities that require the use of ear devices as protection from the loud environment. The wearing of such devices forces workers to stay very close to each other to facilitate conversation among them, and such close working situations also facilitate transmission of meningococci.

Although the relationship between meningococci carriage prevalence and disease incidence is not fully understood, the evidence gathered during this study showed a dominance of the C:23:P1.14–6 phenotype strain among workers from both refineries, reinforcing the concept that the dominance of a particular strain is an important marker of epidemic conditions (*30*,*31*). Also, in accordance with previous findings from other studies, we observed that polysaccharide vaccination had no effect on carriage and did not interrupt transmission to susceptible contacts (*4*,*24*). These results represent a challenge to the current policy of using the meningococcal polysaccharide A/C vaccine to control outbreaks of MenC disease, and they have key implications for future vaccination strategies. Our findings emphasize the need to review such policies and to consider using MCC vaccines rather than meningococcal polysaccharide A/C vaccines to control MenC disease outbreaks.
